# Reconstructing signed relations from interaction data

**DOI:** 10.1038/s41598-023-47822-1

**Published:** 2023-11-24

**Authors:** Georges Andres, Giona Casiraghi, Giacomo Vaccario, Frank Schweitzer

**Affiliations:** ETH Zürich, Chair of Systems Design, Weinbergstrasse 56/58, Zürich, Switzerland

**Keywords:** Complex networks, Mathematics and computing

## Abstract

Positive and negative relations play an essential role in human behavior and shape the communities we live in. Despite their importance, data about signed relations is rare and commonly gathered through surveys. Interaction data is more abundant, for instance, in the form of proximity or communication data. So far, though, it could not be utilized to detect signed relations. In this paper, we show how the underlying signed relations can be extracted with such data. Employing a statistical network approach, we construct networks of signed relations in five communities. We then show that these relations correspond to the ones reported by the individuals themselves. Additionally, using inferred relations, we study the homophily of individuals with respect to gender, religious beliefs, and financial backgrounds. Finally, we study group cohesion in the analyzed communities by evaluating triad statistics in the reconstructed signed network.

## Introduction

Social interactions and signed relations are distinct yet related facets of human behavior. Social interactions are short-lived contacts during which individuals exercise directed or reciprocal influence over one another^1^. Individuals can interact via different means, and their interactions may repeatedly occur over time. Signed relations, such as friendship and enmity, are interpersonal relations characterized by a *sign* (positive or negative) reflecting how one person feels or thinks about another. Signed relations are long-lived and change less frequently as more effort is required to form or change them.

While social interactions and signed relations are different, they are coupled to each other–relations acting as drivers for interactions. A positive relation commonly induces more interactions, while a negative one hinders them^2^. Moreover, humans perceive surrounding patterns of positive and negative relations^3^ to which they adapt^4^. Over time, such adaptations can lead to interactions primarily within cohesive groups, potentially leading to echo chambers  . Negative links may be formed across opposing groups, pushing communities towards segregation and, eventually, polarization^5,6^.

To understand such phenomena quantitatively, we require data on the positive and negative relations, which is rare. Interaction data is, instead, more abundant. However, it does not directly inform us about the relations among individuals. This leads to the problem of inferring meaningful information only from interaction data. Usually, this problem is addressed by taking the network perspective, where nodes represent individuals and edges their interactions^7–11^. Network filtering^12^ and backboning methods^13^ can extract relevant connections from observed noisy interactions and find successful applications in biology^14,15^ and economics^16^. Alternative methods use thresholding rules^17^, take a topic modeling perspective^18^ or use relational event models^19^. All these methods, though, can at most be applied to the study of *unsigned* relations or require knowledge about the exact time-ordering of both interactions and relations. For the recovery of *signed* relations, we require novel approaches. Only a few recent works^20,21^ have developed methods with precisely this goal in mind.

Following this path, we introduce a statistical network method to infer weighted signed relations from a collection of unsigned, repeated interactions. We will refer to it as the $\Phi$-method. It relies on the central assumption that a statistical *over*-representation of interactions signals a *positive* relation and an *under*-representation signals a *negative relation*. This assumption is motivated by the longstanding theoretical argument that individuals with positive relations are more likely to interact^2,22^ and its empirical evidence across different communities^23–25^. Moreover, the idea that negative relation induces fewer interactions is supported by the arguments that individuals avoid others who are considered a source of discomfort rather than pleasure^26–28^. Hence, the $\Phi$-method is the counter-part to methods developed for inferring signed relations from repeated signed interactions^29–31^.

To demonstrate our $\Phi$-method, we utilize five classical interaction datasets of social communities. These are a karate club in a university^32^ (KC), a windsurfer community^3^ (WS), a high school in France^33^ (HS), participants in the Nethealth project^34^ (NH) and user of the Epinions website^35^ (EP). These social communities are chosen because they, in addition to interactions, contain information about social relations that can be used to validate our method.

With our method, we reconstruct the underlying relational networks of the five communities. The inferred signed relations allow us to study pairs and triads of individuals in a new light. We illustrate the strength of having access to the complete relational structure of communities, which we represent using a weighted signed network. To this end, we investigate the pairwise homophily, relational triads, and cohesiveness of groups in the communities. Note that we refer to social communities (KC, WS, HS, NH, EP) rather than those detected by community-detection algorithms.

## Results

### Inference of signed networks

To infer the weighted signed networks $\mathcal S_{i}$ for the five communities KC, HS, WS, NH, and EP (extended details provided in “Methods”), we first construct an interaction network $\mathcal G_{i}$. An edge $e_{v\rightarrow w}$ in $\mathcal G_{i}$ is created every time an interaction between individuals *v* and *w* is observed in the respective dataset. Furthermore, each dataset contains a small set of *reported relations* obtained by surveying a subset of the individuals or using a proxy (e.g., declared trust and distrust in EP). Such reported relations are either binary (i.e., positive/neutral or positive/negative), ordinal (i.e., strong positive, positive, neutral, negative), or continuous (i.e., how strong they are).

**Figure 1:**
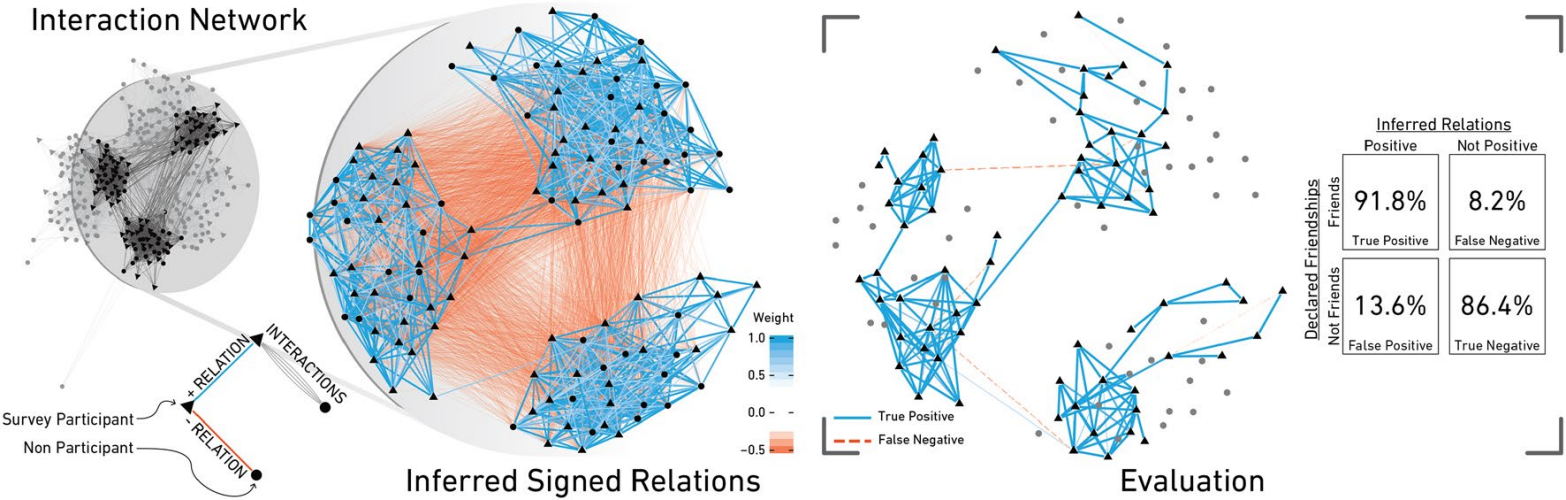
**(Left)** Interaction network $\mathcal {G}_{HS}$ from the HS dataset. Nodes represent individuals, and edges represent recorded interactions between them. Multiple interactions are shown by parallel edges. **(Center)** Inferred signed network $\mathcal {S}_{HS}$ shown only for a subset individuals. Positive relations are represented by blue edges (darker color refers to larger weight). **(Right)** Network of declared friendship relations among individuals. We report a summary of the evaluation in a confusion matrix.

In Fig. 1, we visualize the interaction network $\mathcal G_{\text {HS}}$ only for HS, which records interactions between students in a French high school divided into nine classes. From $\mathcal G_{\text {HS}}$ we infer the weighted signed network $\mathcal S_{\text {HS}}$ using the $\Phi$-method. For each pair (*v*, *w*) of individuals, the weight of the relation $s_{v\rightarrow w}$ is obtained as a linear combination of the probability that two individuals are interacting more than expected with the probability of interacting less than expected (see “Methods” for details). The coefficients of this linear combination are estimated based on the few reported relations in the community. Once determined, this allows us to infer both positive and negative relations between *all* individuals. In^36^, we provide an implementation to quantify the probabilities mentioned above within the R library ghypernet.

In the reconstructed weighted signed network $\mathcal S_{\text {HS}}$, we observe clusters of positive relations with weak negative ties between the clusters. This pattern matches the class separation within the high school. If we compare $\mathcal S_{\text {HS}}$ to the declared friendships provided in the survey (Fig. 1 (right)), we see that most declared friendships are within classes and only a few across classes.

### Accurate prediction of reported relations

Using the $\Phi$-method, we accurately predict the reported relations between individuals. To evaluate this accuracy, we perform both an in-sample and an out-of-sample prediction task where the dependent variable is the reported relation and the predictor the value of $s_{v\rightarrow w}$. We detail the results of the prediction tasks in Table 1. For HS, NH, KC, and EP, the reported signed relations are categorical (friends/not friends, trust/distrust, or individuals feeling a strong positive, positive, neutral, or negative attitude towards others). Hence, we evaluate $\mathcal S_{i}$ by means of standard classification methods and list the resulting sensitivity, specificity, and balanced accuracy (see “Methods”). All these scores are remarkably high and above $80\%$—which holds for both the in-sample and the out-of-sample predictions—for HS, NH, and KC. For EP, the scores are slightly lower but still above $77\%$ except for the specificity. The lower specificity is linked to the limitation of the $\Phi$-method that we elaborate on in the discussion. For WS, the reported signed relations are continuous. Thus, we model them with a linear regression. We evaluate the goodness of fit using the R$^{2}$ and the root-mean-squared-error. These continuous relations are harder to model, as they were obtained through a convoluted interview process. Hence, the reported relations are more noisy. Our goodness of fit suffers from this with an R$^{2}$ just above 0.3.

We find that the $\Phi$-method is robust in handling unseen data. For all datasets, we preserve a very similar accuracy between the in-sample and the out-of-sample prediction. In Table 5 of “Methods” section, we further show that the $\Phi$-method outperforms other approaches for predicting relations based on thresholding rules or network modularity.

**Table 1:** Quality of the model for in-sample and out-of-sample predictions.

	HS	NH	KC	EP	WS
Model specification	Friends $\sim$ $\phi$	Friends $\sim$ $\phi$	Faction $\sim$ $\phi$	Trust $\sim$ $\phi$	Closeness $\sim$ $\phi$
In-sample					
Sensitivity	0.831	0.831	0.931	0.805	
Specificity	0.931	0.985	0.886	0.742	
Balanced accuracy	0.881	0.908	0.908	0.774	
R$^{2}$					0.313
RMSE					0.118
Out-of-sample					
Sensitivity	0.8	0.822	0.818	0.863	
Specificity	0.941	0.986	1	0.689	
Balanced accuracy	0.871	0.904	0.909	0.776	
R$^{2}$					0.331
RMSE					0.117

We report the sensitivity, specificity, and balanced accuracy for HS, NH, KC, and EP. For the continuous relations in WS, we report the R$^{2}$ and the root-mean-squared-error (RMSE). For KC, we map the multi-class prediction task to a binary prediction for consistency. Specifically, we map positive attitudes to a positive relation and neutral and negative attitudes to “non-positive relations.” Overall, the model quality is good for the binary relations and worse for the continuous ones. The model is robust as the out-of-sample prediction only loses little compared to the in-sample prediction.

### Homophily

**Figure 2:**
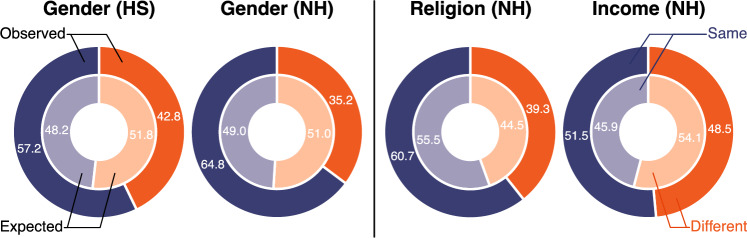
**(Left)** Gender homophily in HS and NH. **(Right)** Religion and income homophily in NH. **(Outer Rings)** Probability in percentage that pairs of individuals with *inferred* positive relations share the same attribute (blue sector) against those with different attributes (orange sector). **(Inner Rings)** Probability of two *random* individuals sharing the same attribute (blue sector) against different attributes (orange sector). A comparison between outer and inner rings shows that all three types of homophily are present, i.e., the outer blue sector is larger than the inner one.

Homophily is the phenomenon of similar individuals being more likely to form positive relations. In the inferred signed networks $\mathcal S_{\text {HS}}$ and $\mathcal S_{\text {NH}}$, we find strong gender homophily, i.e., the specific case in which similarity is defined by gender. To test the presence of this phenomenon, we compare two probabilities (in percentage): (1) the probability that individuals with a positive relation also have the same gender and (2) the probability that randomly sampled pairs of individuals have the same gender. These are shown in Fig. 2 in the outer (1) and inner (2) circles. We only have gender data in the NS and HS datasets, so we restrict the analysis to these two datasets. We find that the probability that individuals with a positive relation are also of the same gender is larger than the reference probability of randomly sampled pairs of the same gender (Fig. 2). Precisely, compared to the reference case, it is approximately $20\%$ and $30\%$ more likely that individuals with a positive relation have the same gender in the HS and NH datasets, respectively. By performing a binomial test, we verify that these results are statistically significant (see “Methods” for details). In Section S1 of the SI, we further characterize the effect of gender on the inferred signed relations.

Apart from gender, we find that religion and parental income homophily are of lesser importance to university students. This is shown in Fig. 2 by comparing 64.8 versus 49.0 for gender to 60.7 versus 55.5 for religion, and 51.5 versus 45.9 for parental income. Only for this dataset do we have such additional information. The probability that friends have similar religious beliefs or parental income is slightly larger than in the reference case but nevertheless significant.

### Beyond dyadic properties

Thanks to our analysis, we have attributed a weighted signed relation $s_{v\rightarrow w} = \phi _{vw}(a,b)$ to each pair of individuals, where $\phi _{vw}(a,b)$ is defined in Eq. (4). The datasets contain additional information about the belonging of these individuals to different groups (e.g., classes and memberships). By looking at triads composed of three individuals, we can now characterize these groups. Considering only the sign of relations, four types of triads $T_{\tau }$ can appear: ($+++$) ($T_{1}$), ($++-$) ($T_{2}$), ($+--$) ($T_{3}$), ($---$) ($T_{4}$). For each triad $t=(v,w,z)$ of a given type $T_{\tau }$, we assign a weight $\omega _{t}$ by multiplying the weighted signed relations $s_{v\rightarrow w}$, $s_{w\rightarrow z}$, and $s_{z\rightarrow v}$^37^. We define group *cohesion* by means of triads $T_{1}$ with three positive relations ($+++$). Group *conflict*, conversely, is defined by those triads $T_{2}$ that have one negative link ($++-$).

Through the weights of the triads, we can quantify the importance of each type of triad for groups (see “Methods” for details). We can distinguish formal groups (e.g., classes) from informal groups. For example the two groups in KC centered around the leaders JA and HI. Analyzing the networks of signed relations $\mathcal {S}_{HS}$, $\mathcal {S}_{KC}$ and $\mathcal {S}_{WS}$, we find that cohesion strongly outweighs conflict only in HS, which contains formal groups. Differently, informal groups emerging in WS and KC show weaker cohesion and a higher presence of conflict. Specifically, Table 2 shows that ($+++$) ($T_{1}$) triads have high importance within the groups of HS (0.98 and 0.96). In the informal groups of WS and KC, their importance decreases to 0.51. Moreover, in the JA group of KC, conflict has almost as much importance as cohesion. Across all analyzed communities, the importance of relational triads with many negative relations, ($+--$) ($T_{3}$) and ($---$) ($T_{4}$), is marginal.

Our analysis of KC further highlights leaders’ influence on group formation. While, at the time of the data collection, KC consisted of a single community, it eventually split into two groups centered around two leaders, JA and HI^32^. Analyzing these two groups separately, we find that the triads *involving* their leaders are strongly cohesive: ($+++$) ($T_{1}$) triads involving HI and JA have an importance of 0.77 and 0.64, respectively (see Table 2 for details). However, when considering triads *not involving* the leaders, we only find cohesion in HI’s group (0.69). JA’s group instead is dominated by conflict (0.53). Hence, we have revealed that the presence of an influential leader is the major characteristic defining the group.

**Table 2:**
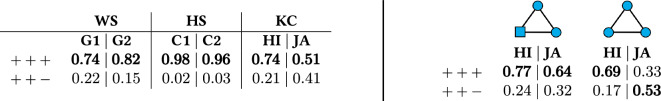
Importance of triad types.

**(Left)** Importance of triad types ($+++$) and ($++-$) for different communities. Each community features groups, and the importance of the triads is calculated within these groups. In all groups but the one of John A. (JA) in KC, the importance of cohesion outweighs conflict. **(Right)** Left are triads in KC involving the leaders of the groups (squared node); right triads not involving the leaders. Mr. Hi’s group is always characterized by cohesion, while John A.’s shows mostly conflict when he is not present.

## Discussion

Our work contributes to the study of human relations by unlocking new applications of interaction data for such investigations. To infer signed relations between individuals, we have employed data about face-to-face contacts (HS), SMS and phone calls (NH), proximity (WS), co-attendance (KC), and online consumer ratings (EP). Traditionally, weighted signed relations are obtained with surveys, an expensive and hardly scalable approach. Instead, interaction data is abundantly available. Despite the different data types, we have shown that our methodology is well suited to extract signed relations. Therefore, social scientists, behavioral researchers, and psychologists can now use interaction data in new ways.

Our central assumption is that positive relations imply more, and negative relations imply fewer interactions. This way of linking interactions to relations is a long-standing assumption in social science^2^, which has been widely tested for positive relations^23–25^. In the case of negative relations, instead, it has rarely been explored, mainly due to a lack of data. The $\Phi$-method fills this gap.

Our broader perspective allows quantifying social phenomena such as homophily, cohesion, and conflict within groups. For instance, we have confirmed that gender homophily is essential in establishing positive relations, such as friendship. Additionally, we have found that leaders can strongly influence the cohesion of a group. This result can be related to the theories of social status and structural balance, according to which individuals adapt their behavior in response to their surroundings^4,38–40^.

The main limitation of this work is linked to the assumption of the $\Phi$-method. It assumes that positive relations imply more and negative relations imply fewer interactions. Even though this is true in many social settings, it is not always true. For instance, in large online social networks, creating a negative relation may require more interactions than retaining a neutral one. Indeed, in this online setting, most users do not know each other, have no relations (i.e., a neutral one), and never interact. Negative relations are instead established between users who interacted negatively once or a few times, leading to negative relations appearing between individuals interacting rather than between individuals not interacting. This process is why, for EP, we obtain a lower specificity than for the other datasets. Another setting in which the assumption of the $\Phi$-method might not hold is in strategic settings where individuals might decide to “keep their friend close, and their enemies closer”.

Overall, our work shows that diverse interaction data can be used to infer signed relations in social communities. The ability to infer signed relations from interaction data enables us to study how relations *evolve* over time. Social theories about structural balance, status, or social impact postulate different mechanisms for *relational changes*. We can now test these mechanisms by leveraging the fine-grained temporal resolution of interaction data. This opportunity paves the way for future research to explore the evolution of signed relations and their effect on communities with an unprecedented resolution.

## Methods

### Data

We require data about social communities containing both interactions *and* declared relations, gathered through surveys. While such data is, in general, scarcely available, we leverage five datasets fulfilling our requirements. They vary in size, number, and type of interactions, and form of surveyed relations. We summarize this information in Table 3.

The data ranges from small communities of under 50 individuals to larger ones encompassing hundreds of people. In these datasets, an interaction $e_{v\rightarrow w}$ indicates ratings, proximity, colocation, or communication events through phone calls, SMS, and WhatsApp between two individuals *v* and *w*. In the three datasets, HS, NH, and EP, interactions were collected automatedly. Thus, they feature the most interactions: up to roughly $4 \cdot 10^{6}$ for EP. In the other two datasets, instead, interactions were recorded manually by researchers. The surveyed relations $r_{vw}$ either indicate a quasi-continuous closeness, attitudes towards some individuals in ordinal categories, a binary friendship, i.e., people being friends or not, or trust/distrust.

**Table 3:** Summary of the main features of the data.

	Nodes	Interactions	Relations (‰ of total)	Directionality	Interaction type	Relation type
HS	327	$67\,613$	406 (7.6‰)	Undirected	Face-to-Face Proximity	Friendship
NH	698	$1\,987\,527$	1353 (2.8‰)	Directed	Communication	Friendship
KC	34	231	36 (64‰)	Undirected	Co-attendance	Ordinal Attitude
EP	$84\,483$	$4\,109\,866$	$689\,728$ (0.1‰)	Directed	Rating	Trust/Distrust
WS	43	1206	903 (1*e*3‰)	Undirected	Proximity	Closeness

#### Windsurfer (WS)

**Figure 3:**
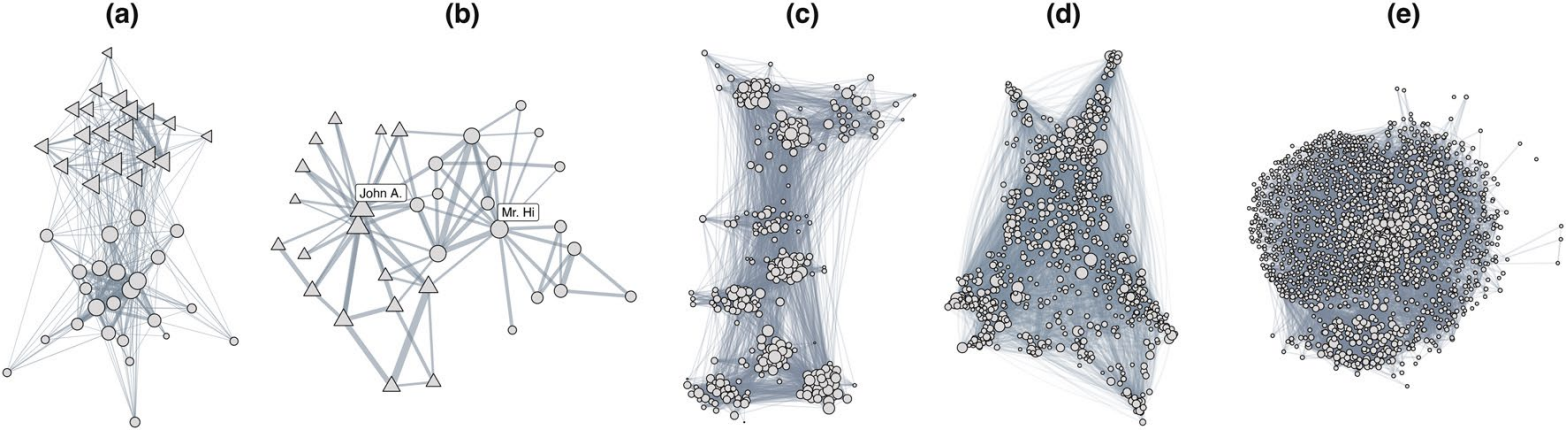
Interaction networks visualized for (**a**) WS, (**b**) the KC, (**c**) HS, (**d**) NH and (**e**) EP. Link weights in the figures are proportional to interaction counts.

The study of the windsurfer community took place in California in the fall of 1986, with the authors being long-time members of this community^3^. The windsurfers were naturally dividing themselves into two groups, newcomers and older members, but there was no display of intergroup conflict. They were observed over 31 days, each day for two 30-min intervals. The interactions can loosely be defined as proximity events, people sitting together for lunch, or social exchanges. Looking at the interaction network (Fig. 3a) makes it clear that most interactions took place within the two informal groups. All community members were interviewed shortly after the conclusion of the observation period. They were asked to perform a sorting task to identify how close they were to each other. This closeness is rescaled to a number in (0, 1) and represents the relations in this dataset. Even though the authors describe a dataset of 54 surfers, only data about 43 of them was released. Differently from all the other datasets analyzed, there are reported relations for *all* pairs. These are shown in Fig. S3a of the SI.

#### Zachary’s Karate Club (KC)

This dataset contains interactions between 34 members of a university karate club over three years. The recorded interactions occurred not during the karate lesson but in different contexts. The karate club had two factions that “were never organisationally crystallized” and “[...] not named” that evolved over time^32^. However, the factions had two leaders: the club president (John. A.) and the karate instructor (Mr. Hi). These factions arose due to a dispute between the leaders over an increase in the costs of lessons. At a certain point, the club split into two clubs, one led by John. A. and the other by Mr. Hi. The club members mainly chose the leader they wanted to join according to the factions they were in before the split^32^. The interaction network (Fig. 3b) makes these factions visible before the split, while inter-faction contacts are still present. Before the split, club members were asked which faction they saw themselves in and whether that sentiment was strong or weak. Only between Mr. Hi and John A. can we assume a negative relation. These declarations form the relations in our analysis (strong positive-, positive-, neutral-, and negative-attitudes). The resulting relations are shown in Fig. S3b of the SI. The data also contains information about each member’s final group after the split.

#### French Highschool (HS)

As a third community, we consider a high school in France. Mastrandrea et al.^33^ have recorded face-to-face interactions between students from four programs and organized them into nine classes. This was done using RFID trackers, which only trigger when individuals are close and facing each other. The interactions are recorded while being at school over five days. Interactions are mainly concentrated within classes, which becomes apparent when considering the network visualization (Fig. 3c). Nevertheless, students interacted with alters from other classes, possibly during breaks. On top of the interactions, information was collected about positive social relations, i.e., friendship. These are shown in Fig. S3c of the SI. The social relations have been collected by means of surveys, as detailed in^33^. Only a subset (41%) of the students had taken active part in the survey. Unfortunately, no information about negative relations was collected.

#### Nethealth Project (NH)

We study the Nethealth Project, a long-lasting (2015–2019) study conducted by the Center for Network Science and Data at the University of Notre Dame^34^. It investigates the social networks and health of initially around 700 undergraduate students, comprising pair-wise interaction data as well as responses to surveys administered in 8 waves over the study period. Interactions were recorded through communication events in the form of in- and out-going calls and messages from the participants’ phones. We construct the interaction network (Fig. 3d) only including people who have at some point participated in the study. The sheer size of the interaction network does not allow us to extract much information from its visualization. However, we see that the degrees of the nodes vary greatly, between 0 at least and 89950 at most. The data contains surveyed friendships, which constitute the relations we use in our work. These are shown in Fig. S4b of the SI. As there were multiple ‘waves’ of surveys, in our analysis, we focus on one wave, namely the second one. This wave contains the most individuals, as subsequently there were some drop-outs. We then only consider interactions happening between the first and second surveys. Our results remain stable over the other waves.

#### Epinions (EP)

Epinions was a general consumer review site where users could create reviews, issue ratings of articles, and establish trust or distrust relations. Interactions are created by rating the article of another user. We limit our prediction task to positive (trust) and negative (distrust) relations, filtering the links where no trust relation was established. As this dataset also contains information about the ratings issued to articles, we employ this information in the prediction task to characterize the authors of articles. Specifically, we use the mean of the received ratings as a proxy for popularity and the standard deviation of the received rating as a proxy for how controversial the author is. Note that we do not use the actual ratings, as this would defy the purpose of using the interactions stripped of their ratings. This leaves us with a dataset as specified in Table 3. In principle, our method allows for a prediction task on all three types of relations, including neutral ones. This comes with a significant loss in accuracy (10–20%), as we cannot a priori distinguish between individuals who did not know each other and those who did. Many different versions of the Epinions dataset exist. We employ the version used in^35^. The size of the interaction network only allows us to plot a sample of it in (Fig. 3e). In Fig. S4a of the SI, we further show the signed network obtained from the trust values.

### The $\Phi$-method

The $\Phi$-method relies on the central assumption that over/under-representations of interactions signal positive/negative relations, a longstanding hypothesis in social sciences^2^. To quantify these over-and under-representations, we compare the observed interaction counts between individuals to a network null model, the hypergeometric ensemble of random graphs (HypE)^41^. By employing a network null model, we define an expectation for the number of interactions between individuals. This expectation should account for all factors that bias the observed number of interactions beyond the effect of signed relations^11^. In this work, we specifically account for the heterogeneity in the activities of the different individuals. That means we account for the fact that a very active individual is more likely to interact with others regardless of whether they share a positive or negative relation. Similarly to a standard configuration model^42^, HypE allows explicit modeling of such heterogenous activities and enables the estimation of network- and dyadic- sampling probabilities through closed-form expressions^41^. It does so by modeling the network generation as a sampling process without replacement from a carefully designed urn.

The urn is filled with a given number of balls, each representing a possible directed edge between two nodes *v* and *w*. An edge $e_{v\rightarrow w}$ from *v* to *w* is considered to be in this set of possible edges if the nodes have non-zero in- and out-degrees $k^{out}_{v}$ and $k^{in}_{w}$, respectively. To account for the different levels of activity of different individuals, we specify the maximum number $\Xi _{vw}$ of possible edges between each pair of individuals to be proportional to the activity—i.e., degree—of each individual in the network. To do so, we define a matrix $\pmb {\Xi }$, whose entries $\Xi _{vw}$ are given by $k^{out}_{v}k^{in}_{w}$. It directly follows that $\sum _{vw} \Xi _{vw} = m^{2}$ is the total number of possible edges and, thus, the number of balls in the urn. A network realization $\pmb {X}$ with *m* edges is given by sampling *m* balls from this urn without replacement. This sampling procedure is akin to hypergeometric sampling, and the probability of finding the observed network configuration $\pmb {A}$ is given by:

1 $$\begin{aligned} \Pr \left( \pmb {X} = \pmb {A}\right) = \frac{\prod _{vw}\left( {\begin{array}{c}\Xi _{vw}\\ A_{vw}\end{array}}\right) }{\left( {\begin{array}{c}m^2\\ m\end{array}}\right) }. \end{aligned}$$

Equation (1) defines HypE, the network ensemble that we use to estimate the pair-wise over-and under-representation of interactions. This ensemble has the benefits of incorporating interdependencies between pairs of individuals, preserving individuals’ activity and attractiveness, and being analytically tractable. For more details, we refer to^41^. While in this work, we focus only on incorporating the *activity* of individuals into our null model, it is, in principle, possible to extend the null model to account for more complex factors, e.g., block or sub-group structures^43^. In Section S2 of the SI, we discuss the role and the effect of such extensions.

From Eq. (1), we extract the two marginal probabilities $P(X_{vw}<A_{vw})$ and $P(X_{vw}>A_{vw})$, where $A_{vw}$ is the observed number of interaction between *v* and *w* and $X_{vw}$ is an hypergeometric random variable:

2 $$\begin{aligned} \Pr \left( X_{vw} < A_{vw}\right)&= \sum _{a_{vw}=0}^{A_{vw}-1}\frac{\left( {\begin{array}{c}\Xi _{vw}\\ a_{vw}\end{array}}\right) \left( {\begin{array}{c}m^2 - \Xi _{vw}\\ m - a_{vw}\end{array}}\right) }{\left( {\begin{array}{c}m^2\\ m\end{array}}\right) } \end{aligned}$$

3 $$\begin{aligned} \Pr \left( X_{vw} > A_{vw}\right)&= \sum _{a_{vw}=A_{vw}+1}^{\Xi _{vw}}\frac{\left( {\begin{array}{c}\Xi _{vw}\\ a_{vw}\end{array}}\right) \left( {\begin{array}{c}m^2 - \Xi _{vw}\\ m - a_{vw}\end{array}}\right) }{\left( {\begin{array}{c}m^2\\ m\end{array}}\right) } \end{aligned}$$

Intuitively, when the first probability is high, it is unlikely to find as many interactions as we observed, indicating an over-representation^11,44^ and, therefore, a positive relation. The same reasoning holds for the second probability, indicating a negative relation. Extending the approach of^20^, we construct the signed relations by taking the difference of these probabilities, weighted according to some constants in what we call the $\Phi$-method $\mathcal {M}_\Phi$:

4 $$\begin{aligned} \phi _{vw}(a,b) = aP(X_{vw}<A_{vw}) + b P(X_{vw}>A_{vw}) \end{aligned}$$

As shown in the following, we can learn the community-dependent constants *a* and *b* when we have access to data about the relations between a small number of individuals in the community. In the Section S3 of the SI, we explore the impact of *a* and *b* in the absence of such training data.

### Training $\Phi$ on data

Whenever we can access data about interactions *and* relations between *some* individuals, we can train the $\Phi$-method to find optimal parameters $\hat{a}$ and $\hat{b}$ to infer signed relations. By extrapolating the learned parameters to *all* pairs in the community, we compute Eq. (4) and construct full signed networks from only a few reported relations.

We employ simple machine learning techniques to estimate the parameters in Eq. (4). Our aim is to classify the reported relation $r_{vw}$ based on the value of $\phi _{vw}(a,b)$:

5 $$\begin{aligned} r_{vw} \sim \phi _{vw}(a,b) + c. \end{aligned}$$

To deal with the different types of relations $r_{vw}$ in our datasets, we must choose the correct classification model and representation of the dependent and independent variables to fit equation Eq. (5). For HS, NH and EP, we have binary relations, $r_{vw} \in \{\text {Friend}, \text {Not Friend}\}$ or $\in \{\text {Trust}, \text {Distrust}\}$, and we perform the classification in Eq. (5) by means of a logistic regression. In KC, multiple ordered categories are possible as individuals declare strong or weak belonging to a faction, $r_{vw} \in \{\text {Strong Positive Attitude}, \text {Positive Attitude}, \text {Neutral}, \text {Negative Attitude}\}$, and, hence, we employ a *cumulative link method*^45^ that results in a ordered multi-class regression. For the continuous relations in WS, $r_{vw}$ refers to some ‘closeness’ $\in (0,1)$, and hence we have a regression rather than a classification problem. To account for this, we employ a linear regression but will still refer to this as a classification task for simplicity.

To compute $\phi _{vw}(a,b)$, we have to consider whether the interaction data is directed or not. EP and NH have directed interactions and relations, so we extract $\phi$ from the directed HypE, see Eq. (2) and Eq. (3). This means that for each pair of individuals, we have two relations to predict and two $\phi$-values to do so. In the undirected datasets (HS, WS, KC), we employ the undirected version of HypE as defined in^41^. Hence, for each pair of individuals, we obtain one $\phi$-value and predict their relation.

Also, as shown in Table 3, we only have partial information about the relational networks, which impacts how to fit Eq. (5). For KC, EP, and WS, we have values, e.g., trust, distrust, or closeness, for the relations $r_{vw}$ between *some* pairs *v*, *w* of individuals. Hence, we use these *known* relations to train our classification model. For HS and NH, only a subset of the individuals participated in the surveys that provides data about social relations. Therefore, only for them do we know whether there is a positive relation (Friend) or not (Not Friend). We train the classification model based on these known relations among the survey participants.

The classification just described gives us estimates $\hat{a}$ and $\hat{b}$ for the parameters in Eq. (4), obtained for the subset of individuals for which reported relations $r_{vw}$ exist. With these, we can extrapolate our findings to the whole community, generating the signed network $\mathcal S_{}$, whose links $s_{v\rightarrow w} = \phi _{vw}(\hat{a},\hat{b})$. In Table 4, we report the coefficients estimated for all datasets. These coefficients are community-dependent. However, *a* is always positive, and *b* is always negative. This finding is aligned with the assumption that having a high over-representation in interactions increases the probability of having a surveyed friendship. Similarly, having a high under-representation decreases this probability. $|\hat{b}|$ is smaller than $|\hat{a}|$ for most datasets, indicating the presence of weak negative links. This observation is connected to the fact that negative links are less represented in the signed relation data. The percentage of negative links in the data varies between 0 and 13.5%. The low values of $|\hat{b}|$ reflect this. The only case where $|\hat{b}|$ is larger than $|\hat{a}|$ is for EP. Additionally, in KC, we observe a large negative $\hat{b}$ compared to the remaining datasets. This is unsurprising as conflicts characterize both the EP and KC communities.

The coefficient *c* in Eq. (5) provides a baseline from which the value of $\phi _{vw}(a,b)$ can be related to the reported relations. Thus, we do not employ such value in constructing the signed network $\mathcal S_{}$.

**Table 4:** Estimated coefficients $\hat{a}$ and $\hat{b}$ for over- and under-representation.

coefficient	predictor	HS	NH	KC	EP	WS
$\hat{a}$	$P(X_{vw}<A_{vw})$	4.71	5.67	2.49	0.66	0.18
$\hat{b}$	$P(X_{vw}>A_{vw})$	− 0.21	− 0.64	− 0.81	− 3.30	− 0.07

$|\hat{b}|$ is smaller than $|\hat{a}|$ for most datasets, indicating the presence of weak negative links.

### Evaluating the $\Phi$-method

#### Scalability

The $\Phi$-method aims at evaluating signed relations for each node pair. That means that there are $\nu ^2$ pairs in a directed network–where $\nu$ is the number of nodes–that need to be analyzed. By choosing HypE as a network ensemble, we can express the marginal probabilities needed to compute Eq. (4) in a closed form. Hence, the complexity of Eq. (4) scales linearly with the number of pairs in the network. This would not be the case when employing network ensembles for which closed-form marginals are not known (e.g., the standard configuration model).

#### Quantifying the quality of the model

Sensitivity, specificity, and balanced accuracy are defined as follows:

6 $$\begin{aligned} \text {Sensitivity} = \frac{TP}{P}, \qquad \text {Specificity} = \frac{TN}{N}, \qquad \text {Bal. Acc.} = \frac{\text {Sensitivity} + \text {Specificity}}{2} \end{aligned}$$

where *TP* and *TN* are the true positives and negatives respectively, and *P* and *N* are the total observed positives and negatives. We perform this classification both in- and out-of-sample. The in-sample classification uses all the available data. For the out-of-sample prediction, we split the data between train and test. The train/test-split was done by randomly sampling $70\%$ of the links. For the EP, NH, and HS, we employ a 10-fold, repeated cross-validation on the train data to compute the parameters $\hat{a}$ and $\hat{b}$. Then, we evaluate the performance on the test data using Eq. (6). For the two small datasets, WS and KC, we perform a Leave-One-Out Cross-validation on the training data. The performance is again evaluated on the test data, using, however, the R-squared and RMSE in the case of the WS.

#### Comparing $\Phi$ to other methods

In the following, we show that the $\Phi$ method outperforms two other methods used to infer relations. The first is a threshold method $\mathcal {M}_{T}$. The user defines a threshold for the interactions over which individuals are assumed to be friends. Similarly, they are assumed to be enemies below this threshold. We assume one threshold for all pairs in the community, and this threshold can be learned from the known relations. Specifically, we use as a predictor the interaction counts $A_{vw}$ in the regression methods:

7 $$\begin{aligned} r_{vw} \sim \alpha A_{vw} + c. \end{aligned}$$

This method disregards any heterogeneities in the individuals, their different levels of activity in the community, or their popularity. We can partly alleviate this by factoring in the degrees of the individuals when defining their relations. By quantifying the expected number of interactions between two individuals based on their degrees, we reach a formulation akin to the one used in the well-known network modularity^46,47^. We call this model the modularity method $\mathcal {M}_{M}$. Formally, it can be written as follows (for directed networks):

8 $$\begin{aligned} \mu _{vw} = A_{vw} - \frac{k_{v}^{out}k_{w}^{in}}{m} \end{aligned}$$

In the undirected case, total degrees are substituted $k_{v}^{out}=k_{v}$ and $k_{w}^{in}=k_{w}$ and the right-hand side is divided by two. While the modularity method now partly accounts for heterogeneities, it disregards that the two individuals we study are part of a larger system, namely the whole network. To compare it to the $\Phi$-method, we use this $\mu _{vw}$ as a predictor in the regression to learn appropriate scaling parameters.

Below, we demonstrate that our proposed $\Phi$-method outperforms both the threshold and the modularity methods in identifying the known relations. To do so, we perform cross-validation on a training subset of the data and validate the learned representations of the relations on a separate testing subset. This out-of-sample prediction task tests the different methods’ ability to predict relations in unseen data based on its learned specification.

In Table 5, we report our findings for all datasets. For the four datasets with categorical relations (HS, NH, KC, EP), we are interested in correctly identifying the known relations, i.e., the true positives and true negatives. Additionally, we are dealing with unbalanced data, where most pairs have no relation. Therefore, we report the balanced accuracy (BA) score, the mean of sensitivity and specificity, which fits our problem best. We report the R$^2$ coefficient for the continuous relations in WS. Consistently across most datasets, the $\Phi$-method outperforms the other two methods. Note that in the case of the small KC and WS datasets, the specific train-test split impacts the out-of-sample prediction. For the KC, the difference in performance is not significant when averaged over different train-test splits.

**Table 5:** Comparing $\Phi$ to other models. Balanced accuracy/ R$^{2}$ obtained from out-of-sample prediction.

	HS (BA)	NH (BA)	KC (BA)	EP (BA)	WS (R$^{2}$)
$\mathcal {M}_{T}$	0.813	0.869	0.840	0.739	0.204
$\mathcal {M}_{M}$	0.824	0.863	0.892	0.743	0.297
$\mathcal {M}_{\Phi }$	0.871	0.904	0.909	0.776	0.331

### Studying signed networks using $\Phi$

#### Significance of homophily

To evaluate the statistical significance of our results on homophily for NH and HS, we perform a binomial test. Let $m_\text {SG}$ be the number of pairs that share the same gender and $m_\text {DG}$ the number of opposite pairs. The probability of randomly sampling a pair with the same gender from the complete data is then $p=m_\text {SG}/(m_\text {SG}+m_\text {DG})$. If we have *n* friends in total and *l* friends who also share the same gender (success), the p-value of the binomial test is given by:

9 $$\begin{aligned} p = P(Y \ge k) = \sum _{i = l}^{n} \left( {\begin{array}{c}n\\ i\end{array}}\right) p^{i}(1-p)^{n-i} \end{aligned}$$

where *Y* is a random variable. If this probability is low, it is improbable to observe at random as many or more homophilous friends as we do in the data. For the HS, we find a p-value of $p_\text {HS}^\text {G}=1.6\cdot 10^{-6}$. For NH, the p-values are $p_\text {NH}^\text {G} = 3.16\cdot 10^{-95}$, $p_\text {NH}^\text {I} = 1.67\cdot 10^{-6}$ and $p_\text {NH}^\text {R} = 3.70\cdot 10^{-5}$ for gender, income and religion respectively. All p-values are significant ($<0.05$).

#### Importance of triads

Let $T_{\tau = \{1,2,3,4\}}$ be the set of all triads of either one of the four types: ($+++$), ($++-$), ($+--$), ($---$). We quantify the importance of a given triad type $T_{\tau }$ as:

10 $$\begin{aligned} n(T_{\tau }) = \sum _{t \in (T_{\tau })} \omega _{t} = \sum _{t \in (T_{\tau })} \Vert \phi _{vw\in t}\Vert \cdot \Vert \phi _{wz\in t}\Vert \cdot \Vert \phi _{zv\in t}\Vert \end{aligned}$$

The sum runs over all triads *t* in the set $T_{\tau }$. The subscript $vw\in t$ signifies that the link between *v* and *w* is in the triad *t*. Note that we use the absolute value of the $\Phi$-measure. Thus, we consider the weight of the relation when evaluating the importance of a given triad. This way, triads containing mainly weak links will contribute less to the importance.

To obtain a number comparable across communities, we normalize the importance of each triad type over the total importance of all triad types.

11 $$\begin{aligned} I({T_{\tau }}) = \frac{n(T_{\tau })}{\psi } \end{aligned}$$

where $\psi = n_{(+++)} + n_{(++-)} + n_{(+--)} + n_{(---)}$. Such a normalization gives us the relative importance, which is the number we report for the different datasets in Table 2 in the main text.

### Supplementary Information

Supplementary Information.

## Data Availability

All datasets used in this work are publicly available at the links provided below: Highschool: http://www.sociopatterns.org/datasets/. Nethealth: http://sites.nd.edu/nethealth/data-2/. Karate Club: https://rdrr.io/github/statnet/statnet.data/man/zach.html. Windsurfers: https://github.com/schochastics/networkdata. Epinions: https://www.kaggle.com/datasets/masoud3/epinions-trust-network.
