# Supplementary material for: Reconstructing signed relations from interaction data
Source: Sci Rep. 2023 Nov 24;13:20689. doi: 10.1038/s41598-023-47822-1 (PMC10673950; doi:10.1038/s41598-023-47822-1)
Supplement: Supplementary file 1 — Supplementary Information. [file 41598_2023_47822_MOESM1_ESM.pdf]

Supplementary Information
Reconstructing signed relations from interaction data

Georges Andres[†], Giona Casiraghi[‡], Giacomo Vaccario[§], Frank Schweitzer[*]

ETH Zürich, Chair of Systems Design
Weinbergstrasse 56/58, Zürich, Switzerland
[†]*geandres@ethz.ch;* [‡]*gcasiraghi@ethz.ch;* [§]*gvaccario@ethz.ch;* [*]*fschweitzer@ethz.ch*

Contents

S1 Local Features of HS and NH Signed Networks 1

S2 Generalizing the Network Ensemble 2

S3 Choosing Φ-method's coefficients in the absence of training data 4

References 5

S4 Supplementary Figures 6

S1 Local Features of HS and NH Signed Networks

We further characterize the local features of the inferred signed networks by analysing how average degrees vary in NH and HS. Figure S1 allows us to compare how the relations inferred for the two datasets differ across genders. Specifically, we find that in the NH dataset, Males have more negative and less positive relations than Females, on average. However, this is balanced by a lower average absolute weight of negative relations and higher average weight of positive relations. In other words, while the unweighted positive and negative degrees differ across genders, the overall intensity of positive and negative relations are comparable.

Differently from the NH case, the HS dataset provides a more interesting result. While the unweighted positive and negative degrees are similar to the NH case, the average weight of the relations is not balancing them. That means, that in this dataset, we infer more negative relations for Males that are also higher in total intensity.

This brief case study allows us to showcase possible applications of the Φ-method when comparing social relations between individuals across different datasets. Unfortunately, we do not have information about gender (nor about other node metadata) for the remaining three datasets. Therefore, we are unable to perform the same analysis for these.

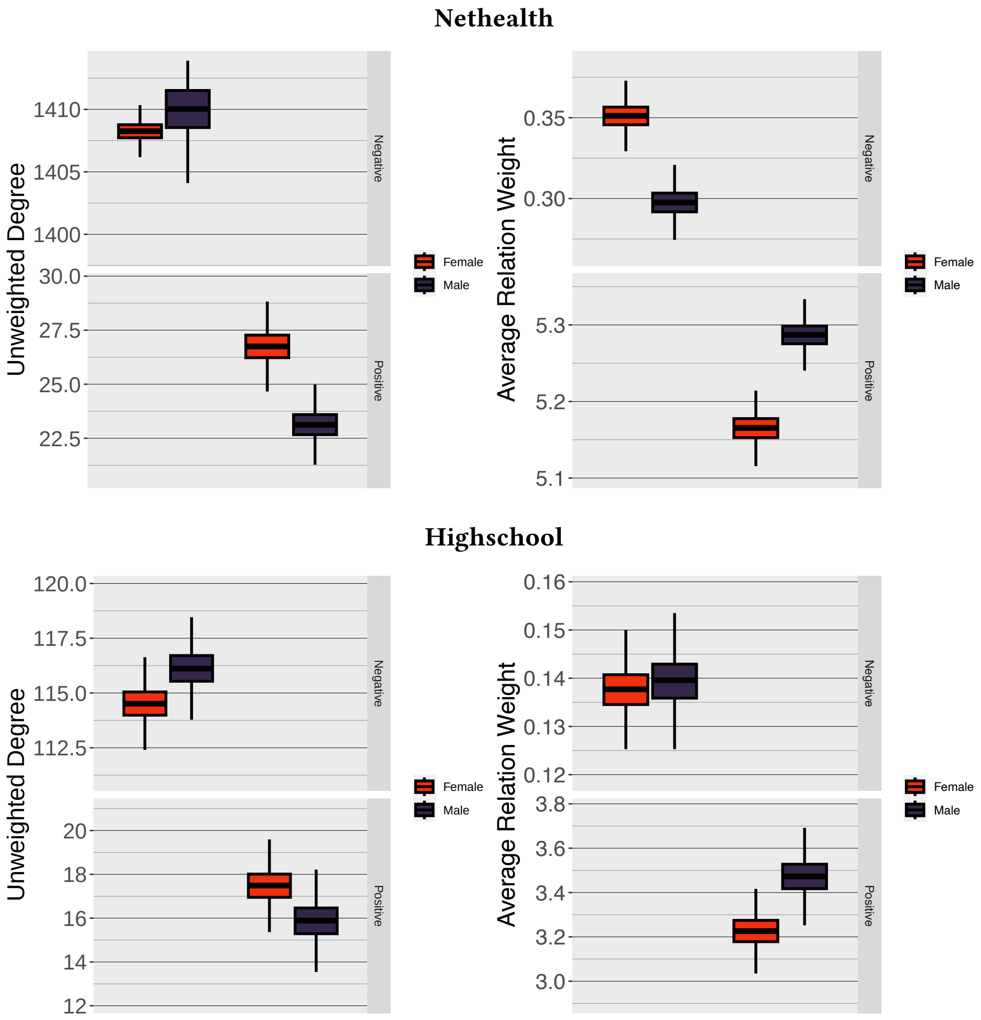

Figure S1: Bootstrap distribution of the average signed degrees stratified by gender in the NH **(Top)** and HS **(Bottom)** datasets. **(Left)**Average unweighted signed degrees. **(Right)** Average weight of the signed relations. A comparison of the left and right plots allows to understand how signed relations are distributed across genders. All bootstrap distributions have been obtained with 10,000 samples.

S2 Generalizing the Network Ensemble

In our study, the identification of signed relations rests on the deviation of edge counts from a specified baseline network null model. Specifically, an over-representation of interactions signifies a positive relation, whereas an under-representation indicates a negative one. Consequently, the choice of an appropriate null model becomes pivotal to delineate this baseline.

Configuration models. A fundamental criterion to define such a baseline is to capture the *activity* of nodes. Neglecting this aspect could misleadingly assign a very active node with solely positive relations to its neighbors. To this end, the class of 'configuration models' is well-established for reflecting node activity [3].

Configuration models encompass a wide array of models, with subtle differences between them. In the main manuscript, we have chosen to focus on the hypergeometric configuration model (HypE). This model ensures an exact total edge count m while preserving the degree sequences $\boldsymbol{k}^{in/out}$ in expectation. Two core advantages of the HypE model are its analytical tractability and the elimination of the need for simulations to ascertain the probabilities in Eq. (4).

The standard (or micro-canonical) configuration model [3] preserves exactly both m and $\boldsymbol{k}^{in/out}$. However, it lacks a closed-form representation for its cumulative marginal probabilities of this model. Consequently, simulations, which are computationally demanding, would have been necessary, impairing the scalability of our method. The Chung-Lu (CL) model [2], on the other hand, postulates an independent Poisson process for each edge, preserving both m and $\boldsymbol{k}^{in/out}$ only in expectation. Given that our datasets originate from finished experiments—with a known edge count m—the hypergeometric model emerged as a more fitting choice. Moreover, the difference between the HypE and CL configuration models becomes negligible for sufficiently large degrees. As shown in Table S1, although model differences can impact the weights of inferred relations, especially the weaker ones, the prediction accuracy remains largely unaltered when employing the CL instead of the HypE configuration model.

Table S1: Comparing Hype to other Null-Models. Balanced accuracy/ R^2 from in- and out-of-sample prediction of the hypergeometric (HypE), the Chung-Lu (CL) and the block-constrained configuration model (BM). CL gives the same accuracy as HypE for all datasets. When information about groups is available, the block-model yields better prediction accuracy.

	HS (BA)	NH (BA)	KC (BA)	EP (BA)	WS (R^2)
**In-sample**					
$\mathcal{M}_{HG}$	0.881	0.908	0.908	0.774	0.313
$\mathcal{M}_{CL}$	0.881	0.908	0.908	0.774	0.313
$\mathcal{M}_{BM}$	0.884	–	–	–	0.502
**Out-of-sample**					
$\mathcal{M}_{HG}$	0.871	0.904	0.909	0.776	0.331
$\mathcal{M}_{CL}$	0.871	0.904	0.909	0.776	0.331
$\mathcal{M}_{BM}$	0.886	–	–	–	0.462

Beyond configuration models. The versatility of our framework allows the integration of more complex null-models. These can encode additional features influencing interaction abundance and relation formation in communities.

For example, both the WS and HS datasets manifest inherent group structures potentially influencing interactions and relations alike. Note that we have to exclude the KC dataset from this analysis. While the KC dataset is also characterised by a group structure at the end of the data collection, we have to exclude it from our analysis here as the groups coevolved together with the interactions, and thus we cannot disentangle whether the relations between individuals are caused by or directly cause the group formation. A dual hypothesis emerges:

(i) intra-group interactions are more likely than inter-group ones, and (ii) positive relations predominantly form within these groups or classes. To incorporate these (or similar) hypotheses into the Φ-method, we proceed as follows. (i) To reflect the fact that interactions are more likely within groups, we can extend the network ensemble to a block model. This change has the effect of varying the expected distribution of interactions between nodes. One possible choice of block model is the block-constrained configuration model [1]. We choose this model for consistency as it is a generalisation of the HypE model. Alternatives such as the degree-corrected stochastic block model [4] would have resulted in similar outcomes. (ii) To reflect the fact that positive relations are more likely to be generated within groups/classes, we can add to Eq. (5) a categorical variable identifying these groups/classes.

With this change, the model quality (in terms of R^2 and Balanced Accuracy) increases, as shown in Table S1. However, we find that, in some group pairs, having both substantially more *and* substantially less interactions than expected *increments* the probability of being friends. This inconsistency means, that in those group pairs there is some additional, unknown, factor that affects the probability of interactions. E.g., during the short observation period in HS, students of some classes may have been working in groups on specific projects affecting their interaction patterns.

This a good example of omitted variable bias. While the model does improve overall at predicting, the values and signs of the coefficients are biased by the fact that some important variable is missing. This bias leads the block model to underestimate interactions for some specific group pairs. While we believe it would be possible to find other, better predictors to suit these datasets, this requires an in-depth analysis of the systems under study and recovering additional data to quantify the missing variables. The latter is not possible for the datasets analysed, and beyond the scope of this article.

S3 Choosing Φ-method's coefficients in the absence of training data

If we had no information about the signed relations, how could we choose the parameters a and b in Eq. (4)? To answer this question, we explore how changing the values of a and b affects the accuracy in predicting relations in the HS and EP datasets. In Fig. S2, we plot Balanced Accuracy values obtained from the prediction task described in the main text against varying a and b. Given the small size of the HS dataset, we perform an out-of-sample prediction there.

In HS, we observe that the balanced accuracy reaches high values for a broad range of a/b combinations. The main reason for this is the lack of negative links in the training data, which results in a very low effect of the b coefficient. In fact, in the EP dataset where we can train the method on negative data, the optimal range of values for the a and b coefficients is more narrow. Similar results hold for the other three datasets (KC, NH, WS). Where we have more information about negative links, the b coefficient has a stronger impact.

Nevertheless, even though the accuracy does not change excessively in response to small changes in the coefficient values, the resulting signed relations may vary considerably. This highlights the importance of the training step when inferring signed relations. In the absence of data to train the model on, we suggest a symmetric choice of a and b. Across all five datasets, the choice of $a = 1$ and $b = -1$ performs at least as well as the modularity and the threshold methods, and often better. With this choice, however, we are reconstructing the signed relations suboptimally. Therefore, whenever training data is available, we highly suggest using it.

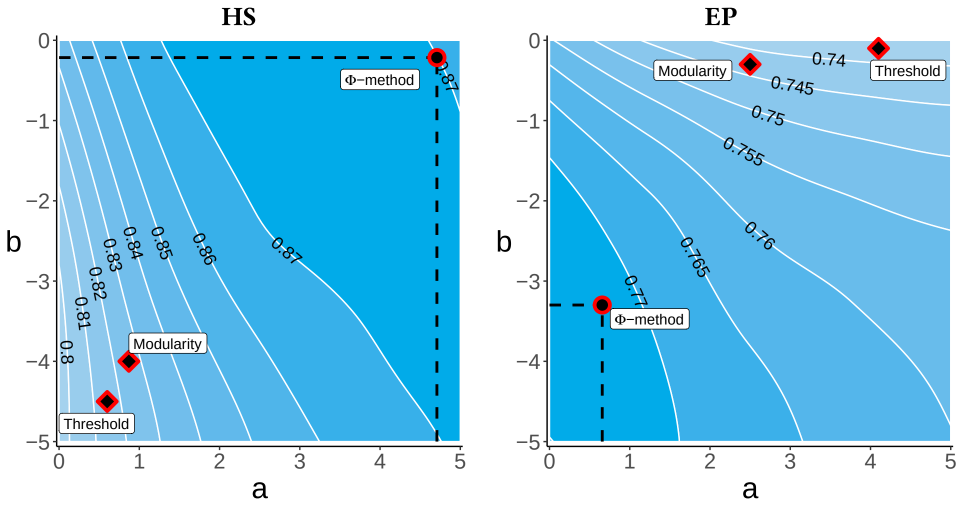

Figure S2: Contour-plot showing the resulting balanced accuracy when varying independently the parameters a and b in Eq. (4) for HS **(Left)** and EP **(Right)**. In accordance with our assumptions, a is positive and b is negative. The red circle shows the optimal model fitted from data at the respective a and b values. The two diamonds show the balanced accuracy for the threshold and the modularity methods placed at the corresponding level, according to the contour-plot. Lighter shades of blue indicate lower accuracy, darker shades of blues indicate higher accuracy values. From the plot it is evident that Φ-method results in very high accuracy values.

References

[1] Casiraghi, G. (2019). The block-constrained configuration model. *Applied Network Science* **4(1)**, 1–22.

[2] Chung, F.; Lu, L. (2002). Connected Components in Random Graphs with Given Expected Degree Sequences. *Annals of Combinatorics* **6**, 125–145.

[3] Fosdick, B. K.; Larremore, D. B.; Nishimura, J.; Ugander, J. (2018). Configuring Random Graph Models with Fixed Degree Sequences. *SIAM Review* **60(2)**, 315–355.

[4] Karrer, B.; Newman, M. E. J. (2011). Stochastic blockmodels and community structure in networks. *Physical Review E* **83**.

S4 Supplementary Figures

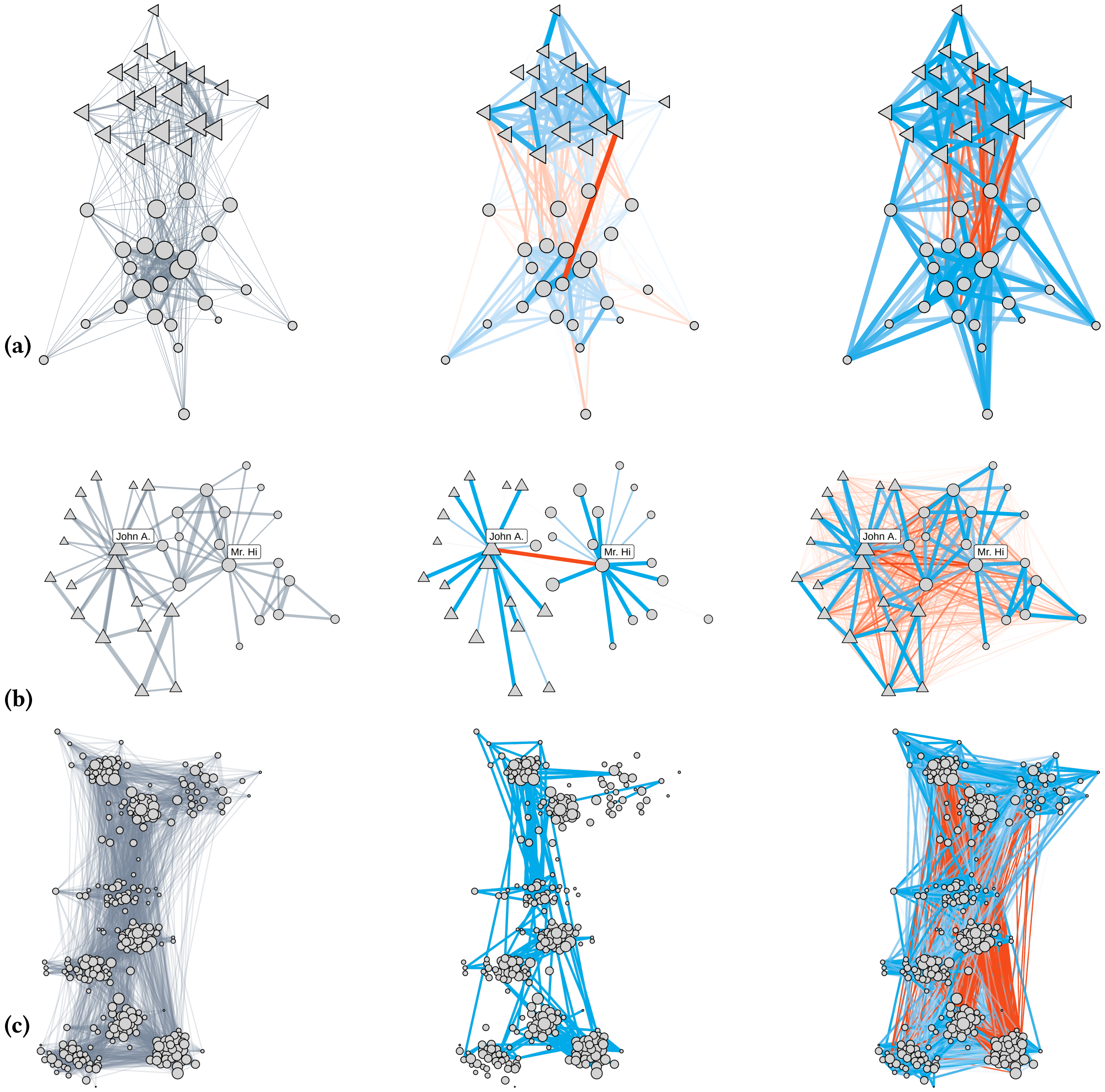

Figure S3: Network plots for the small datasets (a) WS, (b) the KC, (c) HS. (Left column) Interaction networks. The width of edges is proportional to the interaction counts. (Middle column) Networks of reported relations. The colors of the edges in the relational networks reflect the positive (blue) and negative (red) relations reported in the data. For WS these are continuous and we set the threshold between blue and red at the median of the reported relations. In KC, only the leaders have a negative relation, the others have declared either a weak (light blue), a strong (dark blue) or no relation (transparent) to either one of the leaders. The width of edges is proportional to the absolute values of the relations. (Right column) Inferred signed networks. The colors represent the sign of the inferred relation, going from positive (blue), to negative (red). The color scale is asymmetric, such that the strongest red refers to the strongest negative relation and vice versa for positive. The width of the edges is proportional to the absolute value of the inferred relations.

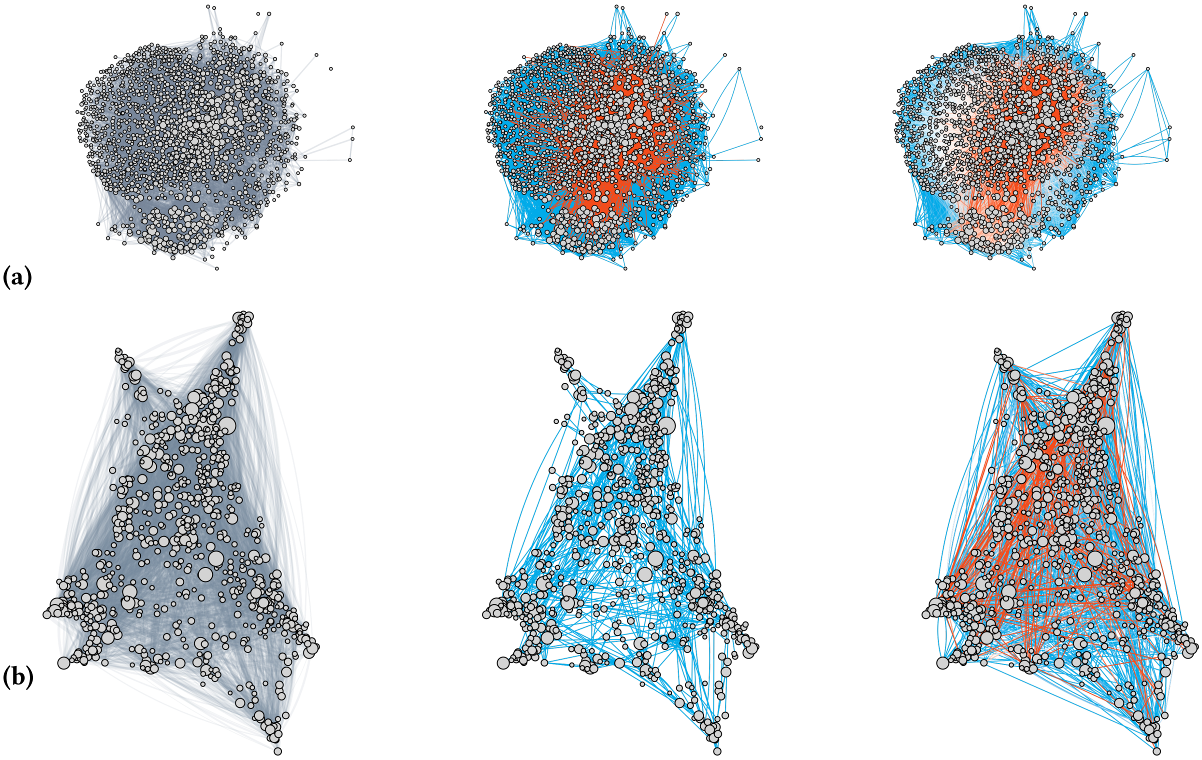

Figure S4: Network plots for the large datasets (a) EP (subsampled), (b) NH. (Left column) Interaction networks. The width of edges is proportional to the interaction counts. (Middle column) Networks of reported relations. The edges in the relational networks reflect the positive (blue) and negative (red) relations reported in the data. (Right column) Inferred signed networks. The colors represent the sign of the inferred relation, going from positive (blue), to negative (red). The color scale is asymmetric, such that the strongest red refers to the strongest negative relation and vice versa for positive. For visualization purposes, all edges have the same width.
